# Microscopic Observation of SARS-Like Particles in RT-qPCR SARS-CoV-2 Positive Sewage Samples

**DOI:** 10.3390/pathogens10050516

**Published:** 2021-04-24

**Authors:** Djamal Brahim Belhaouari, Nathalie Wurtz, Clio Grimaldier, Alexandre Lacoste, Gabriel Augusto Pires de Souza, Gwilherm Penant, Sihem Hannat, Jean-Pierre Baudoin, Bernard La Scola

**Affiliations:** 1 Microbes, Evolution, Phylogeny and Infection (MEPHI), UM63, Institut de Recherche pour le Développement (IRD), Aix- Marseille University, 13005 Marseille, France; belhaouari015@gmail.com (D.B.B.); nathalie.wurtz@univ-amu.fr (N.W.); neogaps@gmail.com (G.A.P.d.S.); hannatsihem@gmail.com (S.H.); 2 IHU Méditerranée Infection, 13005 Marseille, France; 3 Assistance Publique—Hôpitaux de Marseille, 13005 Marseille, France; clio.grimaldier@ap-hm.fr (C.G.); gwilherm.penant@ap-hm.fr (G.P.); 4 Bataillon des Marins Pompiers de Marseille, 13003 Marseille, France; alexandre.lacoste@bmpm.gouv.fr

**Keywords:** SARS-CoV-2 particles, wastewater, Correlative Light and Electron Microscopy, infectious risk

## Abstract

The ongoing outbreak of novel coronavirus pneumonia (COVID-19) caused by SARS-CoV-2 infection has spread rapidly worldwide. The major transmission routes of SARS-CoV-2 are recognised as inhalation of aerosol/droplets and person-to-person contact. However, some studies have demonstrated that live SARS-CoV-2 can be isolated from the faeces and urine of infected patients, which can then enter the wastewater system. The currently available evidence indicates that the viral RNA present in wastewater may become a potential source of epidemiological data. However, to investigate whether wastewater may present a risk to humans such as sewage workers, we investigated whether intact particles of SARS-CoV-2 were observable and whether it was possible to isolate the virus in wastewater. Using a correlative strategy of light microscopy and electron microscopy (CLEM), we demonstrated the presence of intact and degraded SARS-like particles in RT-qPCR SARS-CoV-2-positive sewage sample collected in the city of Marseille. However, the viral infectivity assessment of SARS-CoV-2 in the wastewater was inconclusive, due to the presence of other viruses known to be highly resistant in the environment such as enteroviruses, rhinoviruses, and adenoviruses. Although the survival and the infectious risk of SARS-CoV-2 in wastewater cannot be excluded from our study, additional work may be required to investigate the stability, viability, fate, and decay mechanisms of SARS-CoV-2 thoroughly in wastewater.

## 1. Introduction 

At the end of 2019, a novel human coronavirus known as severe acute respiratory syndrome coronavirus 2 (SARS-CoV-2), was identified in Wuhan, China and subsequently spread worldwide. It has had global consequences on public health, economic activity, and society, which are, in many respects, unprecedented and not yet able to be fully assessed [1]. The disease was named Coronavirus Disease 2019 (COVID-19) by the World Health Organization (WHO). To date, more than 120 million cases and more than 2.5 million deaths have been reported worldwide as of 18 March 2021 [2]. It is an extremely contagious disease with a high risk of person-to-person transmission, with notable transmission levels in hospitals and family settings [3,4].

The SARS-CoV-2 infection causes a series of respiratory illnesses, including severe respiratory syndrome, indicating that the virus most likely infects respiratory epithelial cells [5,6]. The common clinical symptoms for COVID-19 in its current state are fever, dry cough, dyspnoea, muscle soreness, headache, anosmia, and fatigue [7]. Additionally, in the early reports from Wuhan, 2–10% of patients with COVID-19 had gastrointestinal symptoms such as diarrhoea, abdominal pain, and vomiting [8]. Many researchers have also shown that SARS-CoV-2 RNA is detectable in the faeces of infected persons [9]. Furthermore, SARS-CoV-2 RNA can be found in stool samples weeks after the infection is no longer detectable in nasopharyngeal swab samples [10]. Several authors have reported that viable SARS-CoV-2 particles can be obtained from stool samples, as evidenced by the cell culture [11,12,13]

These clinical observations imply that wastewater from affected communities may contain the virus. Wastewater is especially useful as an early warning mechanism for disease outbreaks and as a means of improving the efficacy of public health interventions, as previously demonstrated for enteric viruses such as norovirus, hepatitis A virus, and poliovirus [14,15]. Recently, various studies have detected SARS-CoV-2 RNA in wastewater in various countries around the world, including the Netherlands [16], France [17,18], USA [19], Australia [20], China [21], Japan [22], Dubai [23], United Arab Emirates [24], Turkey [25], and Italy [26]. All show that the quantitative detection of SARS-CoV-2 in sewage samples provides an early and global way of monitoring virus circulation in addition to human epidemiological data.

However, little is known about the potential distribution of the virus in the wastewater and its survival in water [27]. It was suspected that SARS-CoV-2 from wastewater may potentially still be infectious [17], but some reports have hypothesized that the infectious potential of sewage samples is negligible [28]. Investigations are thus needed to elucidate the presence of SARS-CoV-2 particles and their viability in wastewater. Thus, in this study, we investigated the possible presence of SARS-CoV-2 particles in Marseille wastewater using correlative light-electron microscopy and checked the viability of the virus.

## 2. Results

### 2.1. PCR Detection of Viruses in Sewage Sample

In concentrated wastewater of the 11th of September, adenovirus, rhinovirus, enterovirus, and SARS-CoV-2 were detected. Other pathogens targeted by the Biofire^®^ Respiratory Panel 2.1 plus were negative, including for coronaviruses (229E, HKU1, NL63, and OC43); human metapneumovirus; human rhinovirus; Influenza A and B; Middle East Respiratory Syndrome (MERS-CoV); parainfluenza 1, 2, 3, and 4; respiratory syncytial virus; Bordetella parapertussis; Bordetella pertussis; Chlamydia pneumoniae; and Mycoplasma pneumoniae.

### 2.2. Immunohistochemistry and Correlative Light and Electron Microscopy (IHC–CLEM)

**Figure 1:**
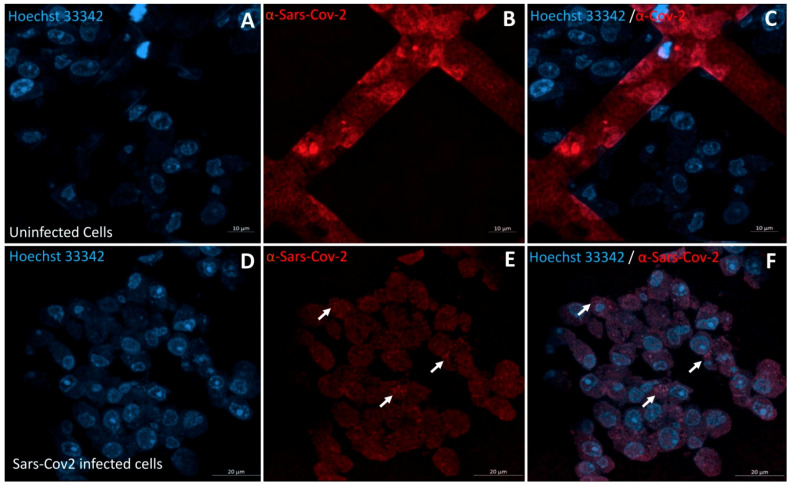
Immunohistochemistry of SARS-CoV-2 in Vero E6 cells. Confocal laser scanning microscope images of 100 nm thick ultra-thin sections (Z-maximal projections) of uninfected (**A**–**C**) and SARS-CoV-2-infected (**D**–**F**) Vero E6 cells: Hoechst 33342 (DNA staining), and anti-SARS-CoV-2 antibody (red). Immunofluorescence staining of SARS-CoV-2 did not show any fluorescence inside the uninfected cells (**B**,**C**) while showing scattered fluorescent spots (red) inside the cells (white arrows) (**E**,**F**). Scale bars: (**A**–**C**) 10 µm; (**D**–**F**) 20 µm.

**Figure 2:**
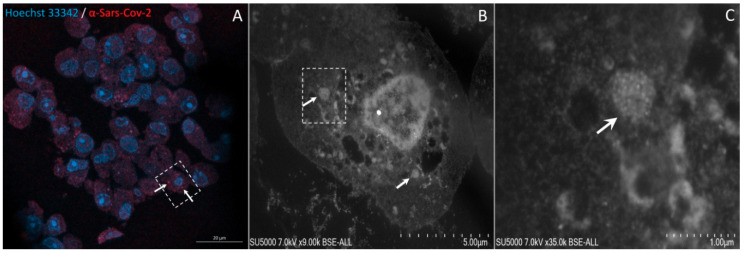
Correlative light fluorescence and electron microscopy of SARS-CoV-2 infected Vero E6 cells. Confocal laser scanning microscopy image of 100-nm-thick ultra-thin section (Z maximal projection) of SARS-CoV-2-infected Vero E6 cells (**A**), with DNA stained with Hoechst 33342 (blue) and SARS-CoV-2 particles labelled with anti-SARS-CoV-2 spike protein (red). Scanning electron microscopy images (**B**,**C**) of the ultra-thin section shown in (**A**). The boxed region of interest in (**A**) is shown at a higher magnification in (**B**). Clusters of SARS-CoV-2 –like particles can be seen in the cell shown in (**B**) (arrows). Boxed region in (**B**) is zoomed in (**C**) showing SARS-CoV-2–like particles (arrow). Scale bars (**A**–**C**) of 20 µm, 5 µm and 1 µm respectively.

The IHC-CLEM was initially standardized on Vero E6 cells infected with a SARS-CoV-2 isolated from patients. In uninfected Vero E6 sections, no SARS-CoV-2 fluorescence signal could be observed (Figure 1B,C), whereas in sections of cells infected with SARS-CoV-2, a fluorescence signal was found (Figure 1E,F). Additionally, the correlation with scanning electron microscopy (SEM) images confirmed that the anti-SARS-CoV-2 immunofluorescence signals coincided with single or brighter clusters of viral particles, inside or outside the cells (Figure 2).

**Figure 3:**
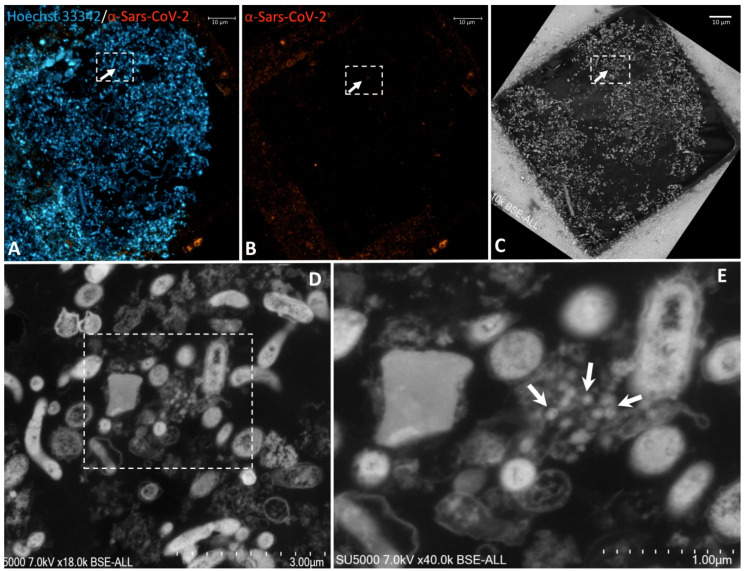
Correlative light fluorescence and electron microscopy in an ultra-thin section of sewage sample. Confocal laser scanning microscopy images of 100-nm-thick ultra-thin section (Z maximal projection) of a sewage sample (**A**,**B**), DNA stained with Hoechst 33342 (blue) and the SARS-CoV-2 particles labelled with anti-SARS-CoV-2 spike protein (orange-red). Scanning electron microscopy images (**C**–**E**) of the ultra-thin section shown in (**A**,**B**). The boxed region of interest in ((**A**–**C**), scale bars 10 µm) is shown at a higher magnification in ((**D**), scale bar 3 µm). Boxed region in (**D**) is zoomed in ((**E**), scale bar 1 µm) with hypo-electron dense circular structures surrounded by a hyper-dense crown-like shapes with 75–100-nm diameters (arrows) are present in the boxed region positive for anti-SARS-CoV-2 fluorescence.

**Figure 4:**
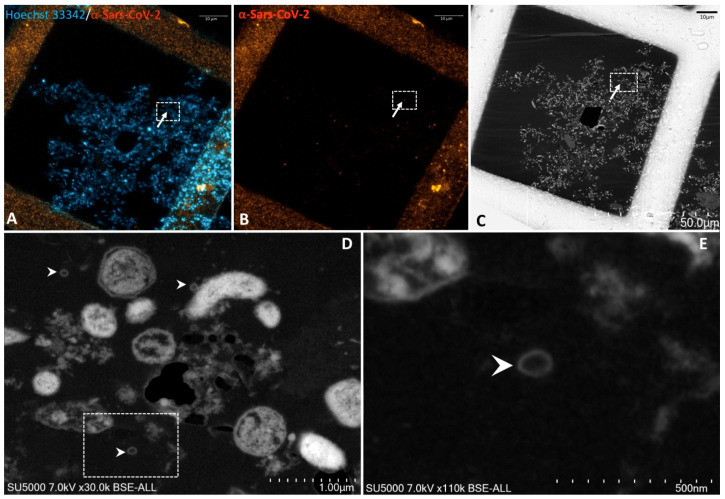
Correlative light fluorescence and electron microscopy in an ultrathin section of sewage sample. Confocal laser scanning microscopy images of a 100-nm-thick ultra-thin section (Z maximal projection) of the sewage sample (**A**,**B**), with DNA staining (Hoechst 33342, blue) and SARS-CoV-2 particles labelled with anti-SARS-CoV-2 spike proteins (orange-red). Scanning electron microscopy images (**C**–**E**) of the ultra-thin section shown in (**A**,**B**). The boxed region of interest in ((**A**–**C**), scale bars 10 µm) is shown at a higher magnification in ((**D**), scale bar 1 µm): SARS-CoV-2 particles appearing empty with only a thin surrounding envelope can be depicted (arrowheads). Boxed region in (**D**) is zoomed in ((**E**), scale bar 500 nm) with empty SARS-CoV-2 like particle (arrowhead).

This approach was then applied to ultrathin sections of wastewater sample. Regions of interest (ROIs) enriched in biological material were identified with the help of Hoechst Dye (DNA staining) (Figure 3A). Laser-scanning of the same regions for excitation of the secondary antibody directed against the anti-SARS-CoV-2 antibody was then performed and revealed scattered fluorescent spots among the material in the wastewater samples (Figure 3B). ROIs with positive anti-SARS-CoV-2 fluorescent spots in the ultrathin sections of wastewater sample previously acquired by Confocal Laser Scanning Microscopy (CLSM) were scanned a second time by electron microscopy after heavy metal contrast. While scanning the sections using SEM, DAPI staining images were conveniently used as a reference for finding regions of interest containing SARS-CoV-2-positive spots (Figure 3A,B). As seen using SEM, anti-SARS-CoV-2 fluorescence spots were localised by SEM in regions containing hypo-electron dense circular structures surrounded by a hyper-dense crown-like shape (Figure 3D,E and Supplementary Materials Figures S1 and S2D,E). Their diameters ranged from 75 to 100 nm. These objects were compatible with the morphology and size of SARS-CoV-2 particles previously described [29,30,31,32]. Furthermore, the fluorescence signal of the anti-SARS-CoV-2 antibody was also found to coincide with regions containing empty SARS-CoV-2-like particles with only a thin surrounding envelope (Figure 4D,E. Supplementary Materials Figure S3D,E).

### 2.3. Infectious Potential of Sewage Samples

**Figure 5:**
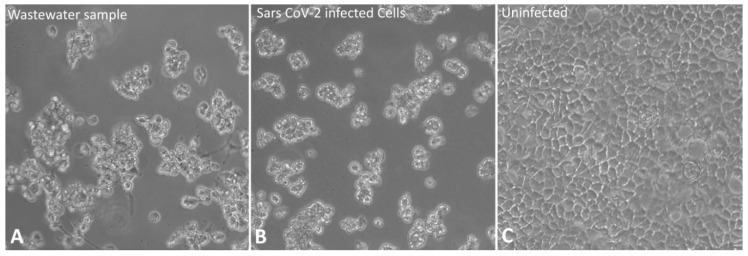
Viral replication assay on Vero E6 cells. Vero E6 cells were inoculated with either a wastewater sample (**A**) or with SARS-CoV-2 ((**B**); positive control) or the culture medium only ((**C**); negative control). A cytopathic effect was observed for both the wastewater sample and SARS-CoV-2 infected cells from two days post-infection.

After one week of culture, no cytopathic effect (CPE) was observed, but after subculture on fresh cells, on the second day post-subculture, the morphology of the Vero E6 cells that comprised the infected monolayer started to display the visual changes, such as cells rounding and cell lysis. The cells lysates agglomerated in large blocks in the supernatant after the destruction of the monolayer on the fifth day post-subculture (Figure 5A). This picture was not evocative of SARS-CoV-2, as observed in the control. Indeed, the evolution of cell infection when infected exclusively by SARS-CoV-2 usually progresses without large clusters of cells lysed at the supernatant. However, it is still possible to observe small sets of cells after the fourth day post infection (Figure 5B). The real-time RT-PCR performed on nucleic acids extracted from the cells inoculated with the concentrated wastewater sample did not detect SARS-CoV-2 targeted the genes, except for rhinovirus, enterovirus, and adenovirus with Ct values of 25, 25, and 23 respectively. This result confirms the presence of these viruses in the wastewater sample as detected using direct detection on the sewage sample. 

**Figure 6:**
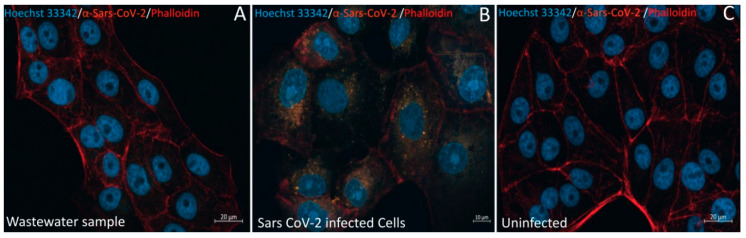
Anti-SARS-CoV-2 immunofluorescence staining on Vero E6 cells. Vero E6 cells were inoculated with either the wastewater sample ((**A**), scale bar 20 µm), SARS-CoV-2 ((**B**), scale bar 10 µm; positive control), or the culture medium only ((**C**), scale bar 20 µm): negative control). Nuclei, F-actin, and SARS-CoV-2 particles were stained using Hoechst 3342 (blue), Phalloidin (far-red), and anti-SARS-CoV-2 antibody (orange-red), respectively. SARS-CoV-2 immunofluorescence staining did not show any fluorescence inside the cells inoculated with the wastewater sample.

The immunofluorescence staining on Vero E6 cells inoculated with the sewage sample did not show a fluorescence signal of the anti-SARS-CoV-2 antibody inside the cells (Figure 6A) contrary to the positive control (SARS-CoV-2-infected Vero E6 cells) (Figure 6B). Importantly, this result rules out the possibility that fluorescent particles identified by SARS-CoV-2 specific antibodies used for IHC-CLEM could be due to cross-reactivity with the three viruses isolated by culture.

## 3. Discussion

In this study, we used a correlative light electron microscopy strategy to detect SARS-CoV-2 particles in sewage from the city of Marseille. This strategy combines the advantages of both light- and electron-microscopy modalities, making it possible to simultaneously locate a target in a comparatively large volume and to determine its ultrastructure [33,34,35]. We used a permeabilization-free immunohistochemistry (IHC) methodology, which is proven efficient screening for viral particles in ultra-thin sections of SARS-CoV-2-infected cells. Such an IHC methodology with no permeabilization for CLEM has also proven efficient for “en bloc” CLEM [36]. Our approach allowed us to get images of SARS-CoV-2 virus-like particles in RT-qPCR SARS-CoV-2 positive sewage samples. Indeed, the objects tagged by anti-SARS-CoV-2 fluorescence appeared by SEM to be compatible in size and shape with those of coronavirus virions previously described [29,30,37].

We observed a cytopathic effect from two days post-inoculation when infecting Vero E6 cells with SARS-CoV-2 RT-qPCR positive purified wastewater samples. However, SARS-CoV-2 RT-qPCR and anti-SARS-CoV-2 immuno-cytochemistry on these cells were negative, suggesting that this cytopathic effect was due to other viruses. Indeed, qRT-PCR revealed the presence of rhinovirus, adenovirus, and enterovirus. Different mechanisms may explain the lack of isolation of SARS-CoV-2. SARS-CoV-2 particles from sewage samples may not be infectious due to being damaged, as was observed on empty SARS-CoV-2-like particles by CLEM. This damage could be due to damage to wastewater matrices, with solvents, detergents, and disinfectants compromising its structural integrity [38].

Moreover, coronaviruses are enveloped viruses with fragile protein spikes that are more prone to alterations by adverse conditions of their structure than to naked viruses [39,40]. Although it has been suggested that SARS-CoV-2 detected in wastewater may remain infectious for hours or even days [17,41,42], to date, no infectious particles have been recovered in Vero E6 cells [28,43]. The WHO has stated that there is no evidence of the survival of SARS-CoV-2 in wastewater or drinking water, as suggested in a technical brief in 2020 [44]. In contrast, it was shown for the previous SARS outbreak in 2004 that SARS-CoV-1 particles could retain their infectious capability when recovered from sewage or wastewater, depending on the conditions [45,46,47]. 

Finally, and although it is unlikely, it cannot be excluded that SARS-CoV-2 particles from the sewage sample may not be isolated because of other competing viruses present in the sample, as we observed in this study. The detection of more environmentally resistant rhinoviruses, adenoviruses, and enteroviruses in sewage water has been previously reported in numerous studies [48,49,50]. The interactions between viruses in the same host can be antagonistic. Several mechanisms have been suggested to mediate viral interference, such as direct blockade of viral entry receptors for one virus by another virus and viral competition for host cell resources [51,52,53].

It should also be considered that the current methods used to concentrate viruses from wastewater, mainly based on ultracentrifugation, which damages fragile spike proteins, may influence the infectious capability of the viruses present in the sample. Indeed, these methods have been developed for concentrating nonenveloped enteric viruses [54]. This limitation has becoming a challenge, and multiple efforts are being made to collect viruses separately and develop an efficient concentration method for SARS-CoV-2 virus from wastewater samples.

In conclusion, in our study, we showed the presence of SARS-CoV-2 viral particles in Marseille wastewater. However, these particles are most probably noninfectious, as particles could appear microscopically damaged and because we were unable to induce SARS-CoV-2 infection in vitro. Nevertheless, the survival and infectious risk of SARS-COV 2 in wastewater cannot be excluded, as other, potentially competing, viruses were also found in wastewater. Thus, efforts are needed to explore wastewater viral ecology for a better understanding of the stability and decay mechanisms of SARS-CoV-2 and better prevent its dissemination.

## 4. Materials and Methods

### 4.1. Wastewater Sampling and Concentration

The wastewater sample was collected from a vacuum sampler that collects the wastewater of Marseille City in France. Sample was collected on the 11th of September 2020 using an automatic sampler “ASP-Station 2000 RPS20B” (Endress Hauser, Huningue, France), then transferred on ice to our laboratory. For concentrations, 200 mL of wastewater was first centrifuged to remove sediments and large particles at 4000× *g* for 30 min at 4 °C. Four 38-mL samples of the supernatant were then centrifuged at 100,000× *g* for two hours using the Sorvall Discovery 90SE ultracentrifuge. Each resulting pellet was then resuspended in 200 µL of PBS, and these suspensions were immediately used for Polymerase-Chain Reaction (PCR), microscopy, and cell inoculation.

### 4.2. RT-PCR for the Quantification of Pathogen RNA in Sewage Samples

To detect viruses in wastewater from a suspension of concentrate, we used a closed system disposable that stores all the necessary reagents for nucleic acid extraction, reverse transcription, and multiplex PCR for the qualitative detection and simultaneous identification of multiple nucleic acids of respiratory bacteria and viruses (BioFire^®^ Respiratory Panel 2.1 plus, BioFire Defense, Salt Lake City, UT, USA). According to the manufacturer’s instructions, this panel was used using the FilmArray Torch instrument (BioMérieux, Grenoble, France). Two hundred microliters of suspension from the concentrated wastewater was tested according to the manufacturer’s instructions, and the interpretation was based on a melt curve analysis.

### 4.3. Resin Embedding and Ultramicrotomy for IHC-CLEM

The suspension from the concentrated wastewater (200 µL) was fixed with 4% paraformaldehyde in 0.1-M sodium cacodylate buffer for five hours at 4 °C. Resin embedding was microwave-assisted with a BiowavePro+ (Pelco, Fresno, CA, USA) (Supplemental Table S1). After rinsing tow times with a mixture of 0.2-M saccharose/0.1-M sodium cacodylate and once with distilled water, samples were gradually dehydrated by successive baths in 50%, 70%, and 96% ethanol. Substitution with LR–White resin (medium grade; Polysciences, Warrington, PA, USA) was achieved by two incubations with a mixture of 100% LR–White resin and 96% ethanol in a 2:1 ratio, two incubations with 100% LR–White resin, and completed with samples in 100% LR–White resin. Resin heat-curing was achieved by polymerization for 72 hours at 60 °C. All solutions used above were 0.2-µm filtered. Ultrathin 100-nm sections were cut using a UC7 ultramicrotome (Leica Microsystems, Wetzlar, Germany) and placed on HR25 300 Mesh Copper/Rhodium grids (TAAB, Aldermaston, England).

### 4.4. Immunohistochemistry and Correlative Light and Electron Microscopy

For anti-SARS-CoV-2 immunohistochemistry (IHC), we used a permeabilization-free procedure. Ultrathin sections were incubated for 30 minutes with BSA 0.1% in H_2_O, with rabbit IgG anti-SARS-CoV-2 primary antibody (Ref. PA5-81795; Thermo-Fischer Scientific, Whaltham, MA, USA) at 1/1000th dilution in H_2_O for three hours, washed twice with BSA 0.1% in H_2_O for five minutes, incubated with secondary anti-rabbit Alexa-647 or Alexa-555 (Ref. A32733; Thermo-Fischer Scientific) secondary antibody at 1/100th dilution in H_2_O for 45 minutes, and washed for five minutes with BSA 0.1% in H_2_O. Sections were also stained with Hoechst3342 (Ref. 62249, Thermo-Fisher Scientific) at 1/1000th dilution in H_2_O for 30 minutes. Finally, sections were washed in H_2_O and air-dried. For all steps above, the incubation was done at room temperature and grids with sections facing down were successively placed on 30-µL drops of respective solutions and were deposited on parafilm in a wet chamber.

### 4.5. Confocal Laser Scanning Microscopy (CLSM) for IHC-CLEM

EM grids were placed on a glass slide with sections facing down and imaged by CLSM on an inverted LSM800 (Zeiss, Jena, Germany) microscope. Acquisitions were performed with ×40 objective and a zoom between 0.5 and 1.7. For the Hoechst3342 stain, Alexa-555 and Alexa-647 fluorophores imaging and 405 nm, 556 nm, and 640 nm lasers were used, respectively. Maximal Z-projections (mean thickness 8 µm) were used to reconstruct signal from the whole sections thickness. The image size was 1024 × 1024 pixels.

### 4.6. Scanning Electron Microscopy (SEM) for IHC-CLEM

After CLSM acquisition, sections were contrasted according to Reynolds [55], attached to double-sided tape mounted on microscopy glass slides with sections facing up and platinum-coated with a MC1000 sputter coater (Hitachi) for 20 s at 10 mA. Observation by SEM of the regions of interest were achieved on a SU5000 scanning electron microscope (Hitachi High-Technologies, Tokyo, Japan) operated in high vacuum at 7 keV with BSE detector and observation mode (spot size 30). Working distance was 6 mm. Capture speed was 40 s. Image size was 1280 × 960 pixels. Magnification ranged between ×180 and ×60,000, with pixel size between 551 and 1.6 nm/pixels, respectively.

### 4.7. Infectious Potential of Sewage Samples

To test the infectious potential of purified wastewater samples that tested positive for SARS-CoV-2 by PCR and CLEM, a Vero E6 (American type culture collection ATCC^®^ CRL-1586™) cell culture model was used. The cells were grown in Dulbecco’s Modified Eagle’s Medium supplemented with 10% foetal bovine serum (FBS) for 2 to 3 days at 37 °C in the presence of 5% CO2. For viral infection, the culture medium was removed, and the cells were inoculated with a filtered concentrated wastewater sample in Minimum Essential Media (MEM) (Life Technologies) supplemented with 4% FBS under previous conditions [56] The CPE was visually monitored by light microscopy every 12 h. To identify growing virus after a CPE was observed, 200 µL of cell culture supernatant was mixed with 200 µL of lysis AVL buffer (Ref 19073 Qiagen, Hilden, Germany). The extraction of viral nucleic acids (DNA and RNA) was performed using EZ1 Virus Mini Kit (Qiagen), following the recommended procedures. The procedure for RT-PCR targeting the SARS-CoV-2 E gene has been detailed elsewhere [57]. For detection of enteroviruses and adenoviruses, the primers, probes, and conditions used were also described previously [58,59]. For the detection of rhinoviruses, the following primers and probes were used: Forward-S1: WGC CVG CGT GGC KGC C, Forward-S2: AGC CYG CGT GGT GCC C, Reverse: GAA ACA CGG ACA CCC AAA GTA GT and probe FAM-CTC CGG CCC CTG AAT GYG GCT AA-TAMRA, at the rate of 300 nm per reaction for primers and 133 nm for the probe. For SARS-CoV-2, rhinoviruses and enterovirus RT-PCR, the LightCycler Multiplex RNA Virus Master kit was used according to the manufacturer’s recommendations (Roche Diagnostics^®^, Mannheim, Germany). For adenoviruses PCR, the DNA Probe Master was used as described by the manufacturer (Roche Diagnostics^®^). All PCR were performed using the LightCycler^®^ 480 Instrument II (Roche Diagnostics^®^).

### 4.8. Immunofluorescence on Vero E6 Cells Inoculated with Sewage Sample

Cells were fixed with 4% paraformaldehyde for 20 minutes and stored in PBS at 4°C until required for immunolabeling. Fixed cells were permeabilized by incubation with 0.1% Triton X-100 for five minutes, blocked by incubation for 30 minutes with 5% normal goat serum (NGS) in PBS, and incubated with primary anti-SARS-CoV-2 antibody (Ref. PA5-81795; Thermo-Fischer Scientific) at 1/1000th dilution for three hours at 28 °C in a humidified chamber. Cells were washed three times with 0.1% Triton ×100 in PBS and then incubated for one hour with secondary anti-rabbit Alexa-647 antibody at 1/100th dilution. Coverslips were then rinsed four times with PBS. Nuclei and F-actin were stained using Hoechst3342 (Ref. 62249, Thermo-Fisher Scientific) and fluorescent phalloidin (Life Technologies, Carlsbad, CA, USA), respectively, with 30-min incubations at room temperature and two washes with PBS and one in distilled water. Vero E6 cells infected with SARS-CoV-2 were taken as positive control and uninfected Vero E6 cells as negative control for experiments. Images were obtained by CLSM with a LSM800 microscope (Zeiss).

## Supplementary Materials

The following are available online at https://www.mdpi.com/article/10.3390/pathogens10050516/s1, Supplementary Figure S1: Correlative light fluorescence and electron microscopy in an ultrathin section of a sewage sample. Confocal laser scanning microscopy images of 100-nm-thick ultra-thin section (Z maximal projection) of a sewage sample (A,B), with DNA staining (Hoechst 33342, blue) and SARS-CoV-2 particles labelled with anti-SARS-CoV-2 spike protein (orange-red). Scanning electron microscopy images (C–E) of the ultra-thin section shown in (A,B). The boxed region of interest in (A–C) is shown at higher magnification in (D). The boxed region in (D) is zoomed in (E) with SARS-CoV-2-like particles (arrow). Supplementary Figure S2: Correlative light fluorescence and electron microscopy in an ultrathin section of a sewage sample. Confocal laser scanning microscopy images of 100 nm thick ultra-thin section (Z maximal projection) of a sewage sample (A,B), with DNA staining (Hoechst 33342, blue) and SARS-CoV-2 particles labelled with anti-SARS-CoV-2 spike protein (orange-red). Scanning electron microscopy images (C–E) of the ultra-thin section are shown in (A,B). The boxed region of interest in (A–C) is shown at higher magnification in (D). The boxed region in (D) is zoomed in (E) with SARS-CoV-2-like particle (arrow). Supplementary Figure S3: Correlative light fluorescence and electron microscopy in an ultrathin section of a sewage sample. Confocal laser scanning microscopy images of a 100-nm thick ultra-thin section (Z maximal projection) of a sewage sample (A,B), with DNA a staining (Hoechst 33342, blue) and SARS-CoV-2 particles labelled with anti-SARS-CoV-2 spike protein (orange-red). Scanning electron microscopy images (C–E) of the ultra-thin section shown in (A,B). The boxed region of interest in (A–C) is shown at higher magnification in (D). The boxed region in (D) is zoomed in (E) with SARS-CoV-2-like particles either appearing full, with an electron-dense core (arrows) or empty with a thin envelope (arrowheads). Supplemental Table S1: Microwave LR–white embedding settings (PELCO BioWave ProPlus).
