# Supplementary material for: Microscopic Observation of SARS-Like Particles in RT-qPCR SARS-CoV-2 Positive Sewage Samples
Source: Pathogens. 2021 Apr 24;10(5):516. doi: 10.3390/pathogens10050516 (PMC8146039; doi:10.3390/pathogens10050516)
Supplement: Supplementary file 1 [file pathogens-10-00516-s001.zip › pathogens-1189744-supplementary/Supplementary table 1.pdf]

| Step | Description | User Prompt (on/of) | time (H:min:s) | Power (Watts) | Temps (C°) | Load Cooler (off/auto/on) | Vacuum/bubbler Pump | SteadyTemp pump (On/off) | SteadyTemp temp (C°) |
|---|---|---|---|---|---|---|---|---|---|
| **1** | 01M Cacodylate/02M Saccharose Buffer | On | 00:00:40 | 250 | 50 | Off | Off | On | 23 |
| **2** | 01M Cacodylate/02M Saccharose Buffer | On | 00:00:40 | 250 | 50 | Off | Off | On | 23 |
| **3** | Water | On | 00:00:40 | 250 | 50 | Off | Off | On | 23 |
| **4** | 50% Ethanol | On | 00:00:40 | 250 | 50 | Off | Off | On | 23 |
| **5** | 70% Ethanol | On | 00:00:40 | 250 | 50 | Off | Off | On | 23 |
| **6** | 96% Ethanol | On | 00:00:40 | 250 | 50 | Off | Off | On | 23 |
| **7** | 96% Ethanol | On | 00:00:40 | 250 | 50 | Off | Off | On | 23 |
| **8** | 2 vol resin 100 % - 1 vol ethanol 96% | On | 00:03:00 | 250 | 50 | Off | Vacuum Auto 20mm Hg 15s/15s | On | 23 |
| **9** | 3 vol resin 100 % - 1 vol ethanol 96% | On | 00:03:00 | 250 | 50 | Off | Vacuum Auto 20mm Hg 15s/15s | On | 23 |
| **10** | Resin 100% | On | 00:03:00 | 250 | 50 | Off | Vacuum Auto 20mm Hg 15s/15s | On | 23 |
| **11** | Resin 100% | On | 00:03:00 | 250 | 50 | Off | Vacuum Auto 20mm Hg 15s/15s | On | 23 |
