# Supplementary figures and images for: Microscopic Observation of SARS-Like Particles in RT-qPCR SARS-CoV-2 Positive Sewage Samples
Source: Pathogens. 2021 Apr 24;10(5):516. doi: 10.3390/pathogens10050516 (PMC8146039; doi:10.3390/pathogens10050516)

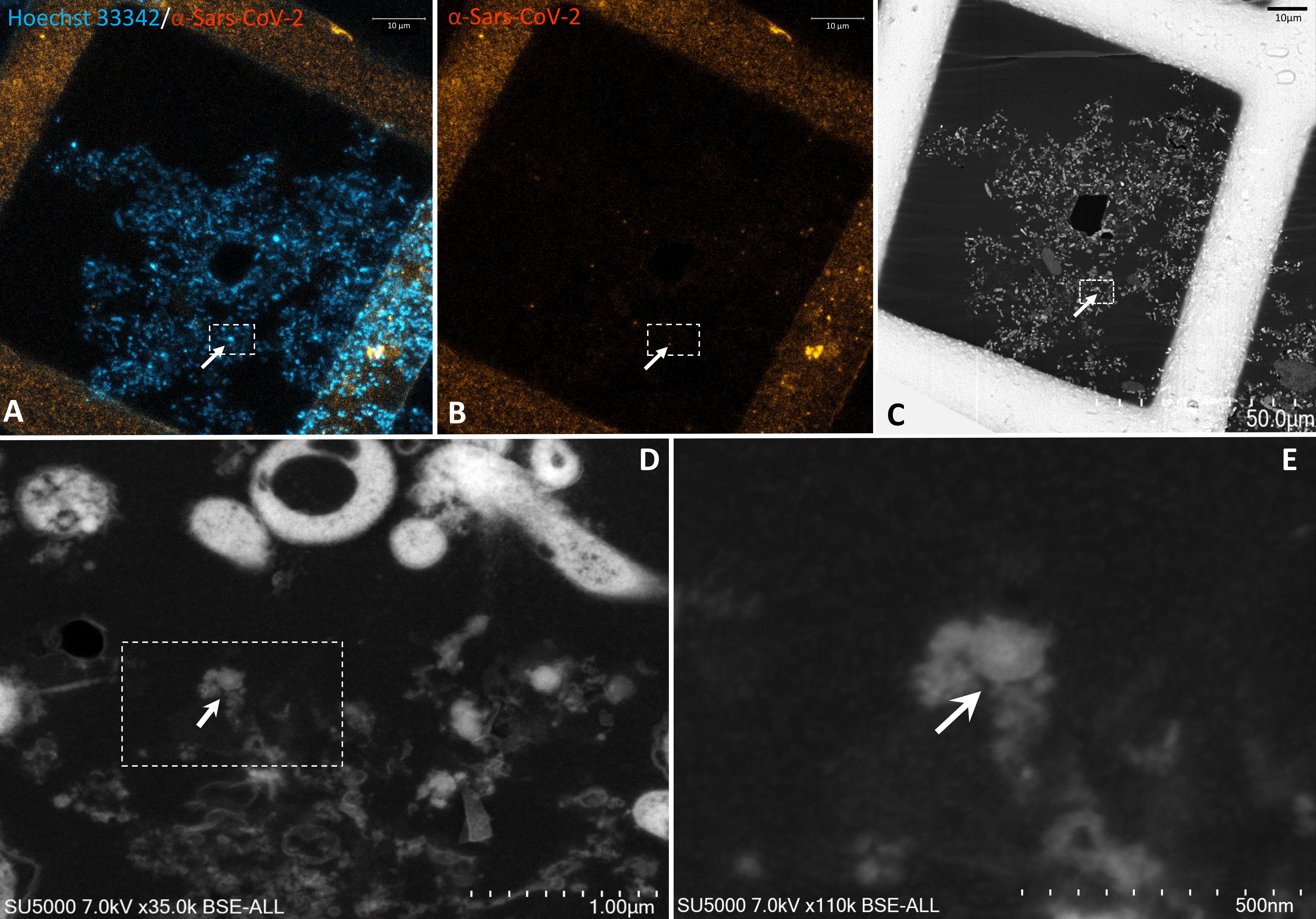

Supplement: Supplementary file 1 [file pathogens-10-00516-s001.zip › pathogens-1189744-supplementary/Supplementary Figure 01.jpg]

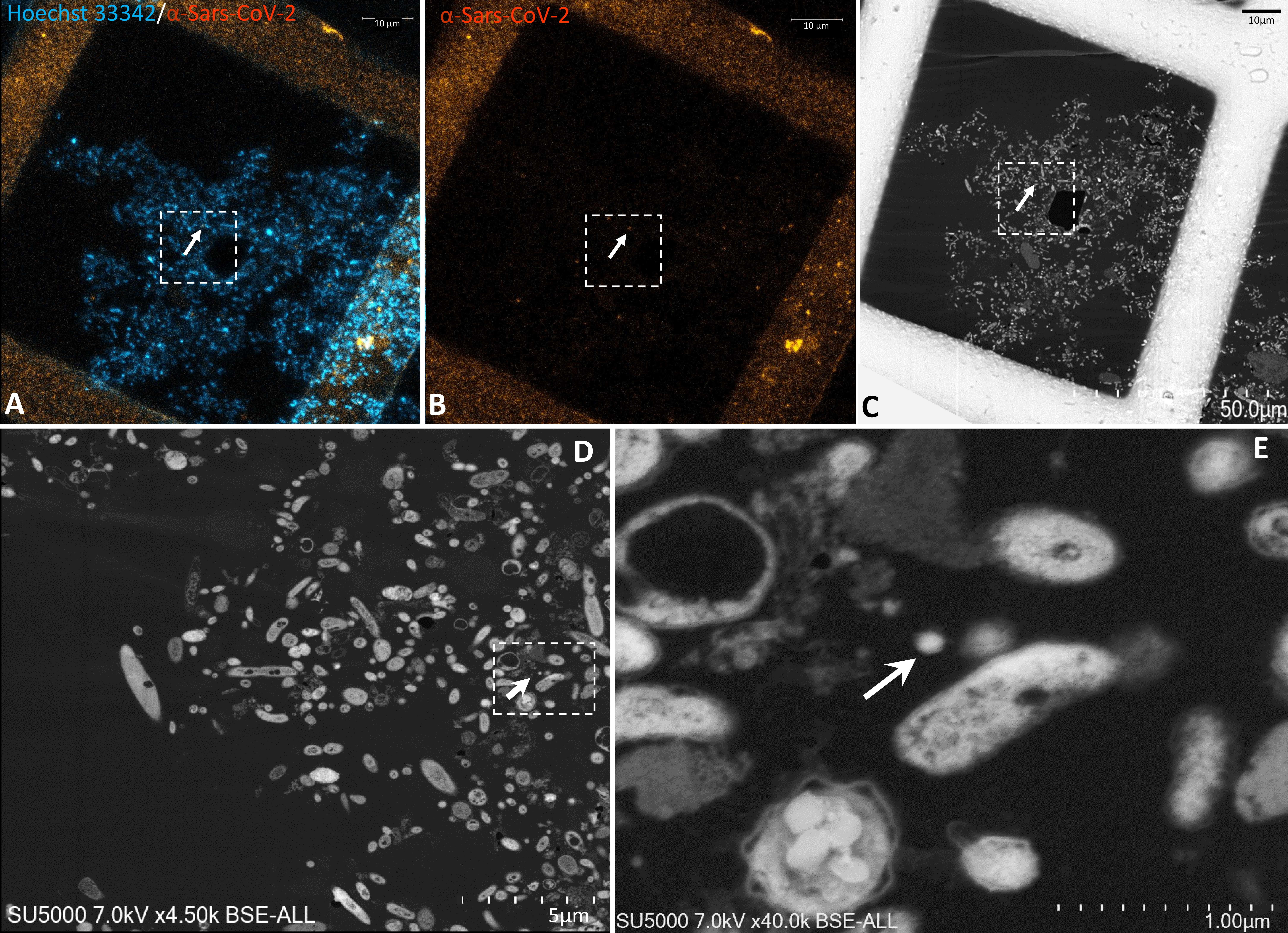

Supplement: Supplementary file 1 [file pathogens-10-00516-s001.zip › pathogens-1189744-supplementary/Supplementary Figure 02.jpg]

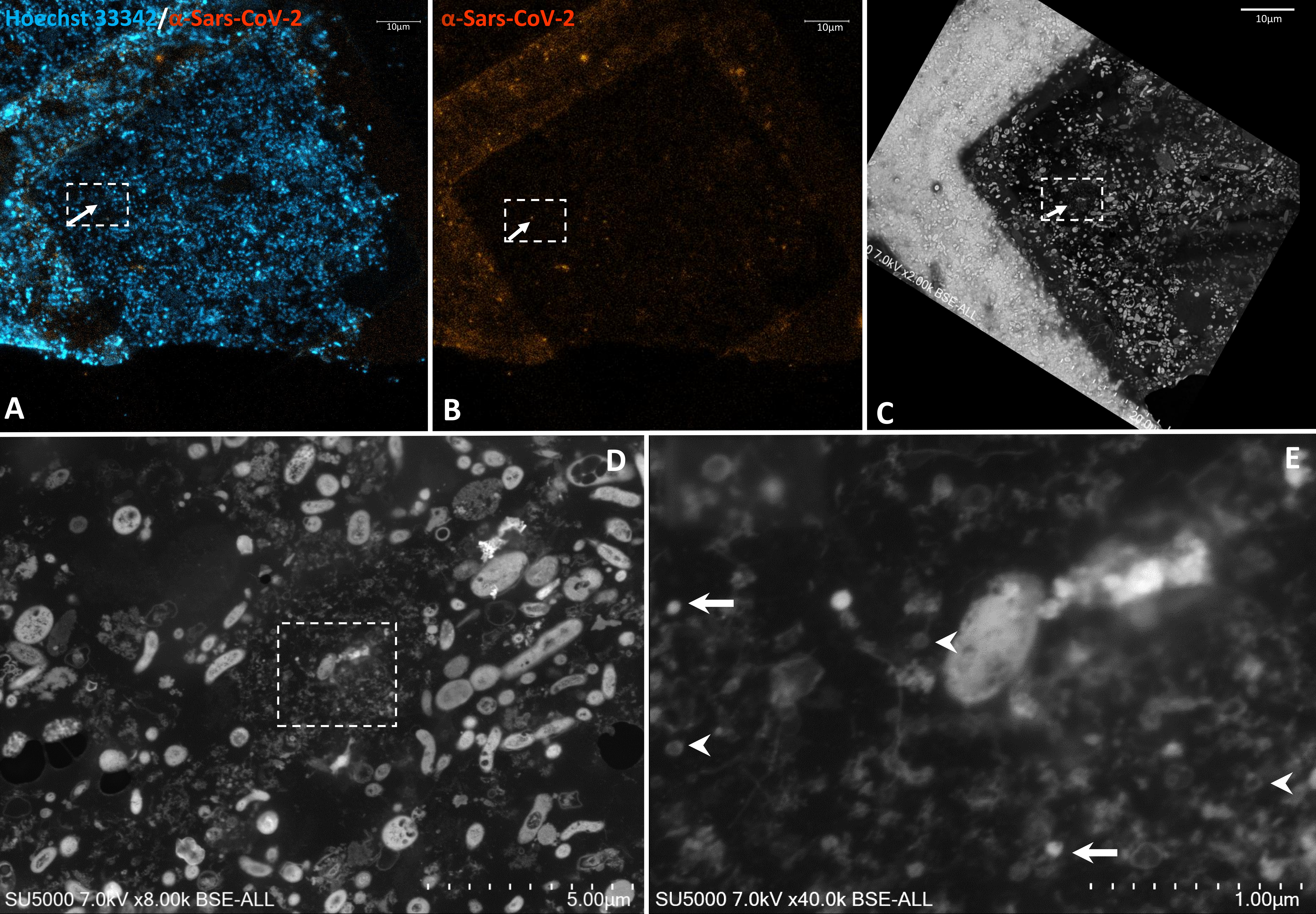

Supplement: Supplementary file 1 [file pathogens-10-00516-s001.zip › pathogens-1189744-supplementary/Supplementary Figure 03.jpg]
